# Cardiovascular mortality and risk behaviours by degree of urbanization before, during and after the economic crisis in Spain

**DOI:** 10.1186/s12889-019-7427-4

**Published:** 2019-08-14

**Authors:** Almudena Moreno-Lostao, Juan M. Guerras, Lourdes Lostao, Luis de la Fuente, David Martínez, Fernando Rodríguez-Artalejo, Enrique Regidor

**Affiliations:** 1 National Epidemiology Center, Instituto de Salud Carlos III, Monforte de Lemos 5, 28029 Madrid, Spain; 2 Department of Sociology, Universidad Pública de Navarra, Pamplona, Spain; 3 CIBER Epidemiología y Salud Pública (CIBERESP), Madrid, Spain; 4 Department of Public Health & Maternal and Child Health, Faculty of Medicine, Universidad Complutense de Madrid, Madrid, Spain; 5 Instituto de Investigación Sanitaria del Hospital Clínico San Carlos (IdISSC), Madrid, Spain; 6 Department of Preventive Medicine and Public Health, School of Medicine, Universidad Autónoma de Madrid/IdiPaz, Madrid, Spain; 7 IMDEA-Food Institute, CEI UAM+CSIC, Madrid, Spain

**Keywords:** Cardiovascular mortality, Risk behaviors, Crisis, Urbanization

## Abstract

**Background:**

To estimate the relationship of the degree of urbanization to cardiovascular mortality and to risk behaviours before, during and after the 2008 economic crisis in Spain.

**Methods:**

In three areas of residence – large urban areas, small urban areas and rural areas – we calculated the rate of premature mortality (0–74 years) from cardiovascular diseases before the crisis (2005–2007), during the crisis (2008–2010 and 2011–2013) and after the crisis (2014–2016), and the prevalence of risk behaviours in 2006, 2011 and 2016. In each period we estimated the mortality rate ratio (MRR) and the prevalence ratio, taking large urban areas as the reference.

**Results:**

In men, no significant differences were observed in mortality between the two urban areas, while the MRR in rural areas went from 0.92 [95% confidence interval, 0.90–0.94) in 2005–2007 to 0.94 (0.92–0.96) in 2014–2016. In women, no significant differences were observed in mortality between the rural and large urban areas, whereas the MRR in small urban areas decreased from 1.11 (1.08–1.14) in 2005–2007 to 1.06 (1.02–1.09) in 2014–2016. The rural areas had the lowest prevalence of smoking, obesity and physical inactivity in men, and of obesity in women. No significant differences were observed in smoking or physical inactivity by area of residence in women.

**Conclusion:**

The pattern of cardiovascular mortality by degree of urbanization was similar before and after the crisis, although in women the excess mortality in small urban areas with respect to large urban areas was smaller after the crisis. The different pattern of risk behaviours in men and women, according to area of residence, could explain these findings.

**Electronic supplementary material:**

The online version of this article (10.1186/s12889-019-7427-4) contains supplementary material, which is available to authorized users.

## Background

The difference in patterns of health and disease between rural and urban areas has not been widely studied. It is usually believed that urban residents have worse health than those in rural areas due to greater exposure to stress, air pollution and higher drug and alcohol consumption [1]. However, in some wealthy countries, the mortality rate is higher in rural than in urban areas [2, 3]. One possible reason is the greater cardiovascular mortality in rural areas observed in different countries such as the United States of America (USA), Canada, Australia and Sweden. This finding has been attributed to a higher frequency of cardiovascular risk factors in rural areas, such as smoking, obesity, physical inactivity, diabetes, hypertension or dyslipidemias [2–6].

In Spain, we do not know whether there are differences in cardiovascular mortality by degree of urbanization [7]. The pattern of cardiovascular mortality in Spain has lagged behind that of other countries, both with respect to the decline of this mortality in the last decades of the twentieth century, and to the behavioural factors affecting cardiovascular risk [8–10], since the smoking and obesity epidemics appeared later than in other wealthy countries [11–13]. Thus, the burden of cardiovascular disease could be lower in rural than in urban areas, since these lifestyles may have spread later among rural residents [14].

On the other hand, various studies have shown a strong relationship between macroeconomic fluctuations and cardiovascular mortality in wealthy countries [15–21]. The decline of mortality accelerates during economic crises and slows down in periods of economic growth [15–19]. In Spain, for example, cardiovascular mortality decreased during the economic crisis of 2008 [20, 21], which was attributed to a greater reduction in risk behaviours due to the decline in personal income, as smoking, obesity or physical inactivity.

A recent study has shown an acceleration of the decline in total mortality during the economic crisis in metropolitan areas of the USA due to a greater reduction in cardiovascular death [22]. However, this could differ in rural areas if the relationship between economic circumstances and mortality were to vary by degree of urbanization. For this reason, the authors proposed to investigate changes in mortality during economic crises in urban and rural areas.

In the European Union, the percentage of people at risk of poverty is higher in rural areas than in urban areas. After the appearance of the economic crisis of 2008, this percentage decreased in rural areas and increased in urban areas, or, as in the case of Spain, this percentage increased less in rural areas than in urban areas [23]. The lower employment rate in rural areas and, therefore, the lower economic activity in rural areas, explains this different impact of the economic crisis according to the degree of urbanization. This fact could have had its correlate in a lower decrease in the prevalence of health risk behaviours in rural areas than in urban areas and, consequently, in a lower decrease in cardiovascular mortality in the former than in the latter.

Spain experienced an important economic crisis that began in 2008 and ended in 2014 [24]. An economic crisis is a business cycle contraction when there is a general decline in economic activity, lasting more than a few months, normally visible in real gross domestic product (GDP). The GDP registered a continued decrease during the last semester of 2008 that caused Spain, for the first time in 15 years, to enter a recession. Accordingly, the objective of this study was to examine the relationship of degree of urbanization to premature cardiovascular mortality and risk behaviours for health in Spain before, during and after the economic crisis.

## Methodology

### Sources of data

The National Institute of Statistics (INE) provided data on the population and number of deaths by 5-year age groups and sex, according to population size in the municipality of residence [25]. We used data for the years 2004 to 2016, the last year with information on deaths. The population data were taken from the population registry (Municipal Register of Inhabitants), and the data on deaths from the mortality registry (Death Statistics by Cause of Death). We selected deaths with codes I00-I99 from the International Classification of Diseases, 10th revision.

We analysed premature deaths because it is usually considered that a large portion of such deaths are due to risk behaviours such as smoking, obesity and low physical activity [26–28]. These behaviours increase the risk of cardiovascular diseases, cancer, respiratory diseases and other health problems. Like most studies, we defined “premature” as a death occurring before age 75 [29, 30]. There is no unanimous criterion in the scientific community regarding the age limit for the calculation of premature mortality. Sometimes other upper limits are used, such as the average age of death or life expectancy. The problem with these last criteria is that the limit varies from 1 year to another and varies among population groups, for example between men and women. We have chosen the majority criterion (deaths in persons less than 75 years), since the choice of a fixed age allows the comparison of the premature mortality rate over time and between different population groups and countries.

The prevalence of risk behaviours for health was estimated from the last three national health surveys, carried out in a representative sample of the Spanish population over 15 years of age in 2006, 2011 and 2016 [31]. Stratified multistage sampling was used. The first-stage units were the census sections, stratified by size of municipality. The second-stage units were the households in each of the census sections selected. The sections were selected within each stratum with a probability proportional to their size. The households in each section were selected with the same probability by systematic sampling, after ordering by size of household. Subjects to be interviewed within each household were selected randomly.

In these surveys, subjects were asked about their tobacco consumption, with the following options: a) daily smokers, b) occasional smokers, c) former smokers and d) never smokers. Those who were daily or occasional smokers were considered to be smokers. Subjects were considered not to engage in physical activity if they declared in the corresponding question that they did no physical exercise, and that their leisure time was spent in sedentary activity: going to the movies, watching television. Body mass index (BMI) was estimated by dividing the reported weight by the square of the reported height. Obesity was defined as BMI ≥ 30 kg/m^2^, according the World Health Organization [32]. It is known that smoking, physical inactivity and obesity are associated with an increase in cardiovascular mortality [33–36]. National health surveys collected several factors that show association with mortality from cardiovascular diseases, such as risk behaviors, socioeconomic status or social support. However, we selected those factors that in previous studies have shown the variation in the prevalence during macroeconomic fluctuations [15, 17, 37, 38].

### Statistical analysis

Many investigations consider rural populations to be those with fewer than 10,000 inhabitants [39, 40]. The concept of rurality varies among researchers or even among planners and decision-makers. Several criteria have been proposed: population size, population density, distance from an urban centre, spatial contiguity, economic activity, proportion of residents commuting to work in an urban centre. Some authors argue that the concept of rural also refers to social and cultural attributes. However, the availability of routine information of these characteristics is rare, apart from the conceptual ambiguity of some criteria and the different meaning of others from one country to another. Therefore, most authors use the criteria we have used in our research: population size. It is a definition that can be easily operationalized and, in addition, allows the comparison of research results made in different countries [39]. Accordingly, in the present study, the size of the municipality of residence was grouped into three categories: fewer than 10,000 inhabitants (rural areas), between 10,000 and 100,000 inhabitants (small urban areas) and more than 100,000 inhabitants (large urban areas). In this last category we included provincial capitals with fewer than 100,000 inhabitants.

In each area of residence we calculated the annual rate of premature mortality from cardiovascular diseases in 2004 to 2016 and the mortality rate for the following three 3-year periods: before the crisis (2005–2007), during the crisis (2008–2010 and 2011–2013), and after the crisis (2014–2016). Specifically, we calculated premature mortality per 100,000 inhabitants-year standardized by age, using the 2013 Standard European Population [41]. We then calculated the mean annual percentage change (mAPC) in the mortality rate in each of the 3-year periods, by segmented linear regression taking as a dependent variable the logarithm of the annual age-standardized mortality rate. The relationship between the area of residence and the rate of premature mortality in each 3-year period was summarized with the age-standardized mortality rate ratio calculated by stratified analysis. The confidence intervals of the mortality rate ratio in small urban area and rural areas were calculated using the variance estimated by the Mantel-Haenszel method. Finally, in each area of residence we calculated the age-standardized percentage of smoking, obesity and physical inactivity in 2006, 2011 and 2016. The relationship between area of residence and these three variables was estimated with the percentage ratio, taking large urban areas as the reference.

## Results

The distribution of the population in the three areas varied little during the study period. About two-fifths reside in large urban areas and one-fifth in rural areas. Deaths in rural areas represented 24% of the total in 2005 and around 20% in 2016 (Table 1 and Additional file 1: Table S1 and Additional file 2: Table S2).

**Table 1:**
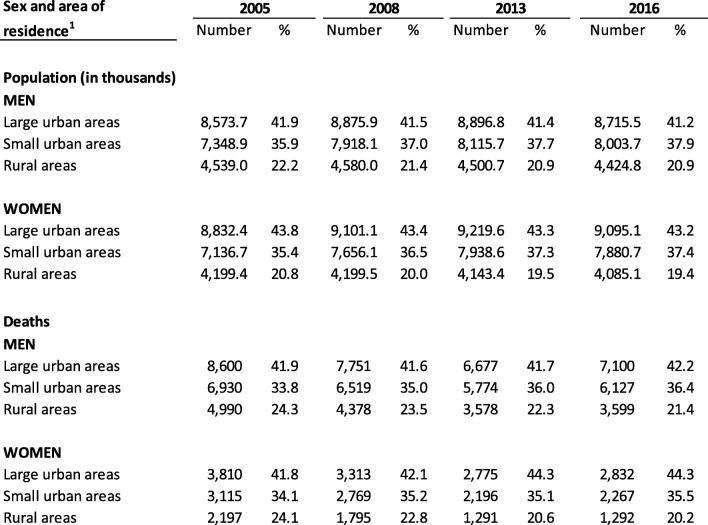
Population and deaths from cardiovascular diseases in people under 75 years of age, by sex and area of residence in various years of the study period

^1^Large urban areas (> 100,000 inhabitants and provincial capitals); small urban areas (10,001 to 100,000 inhabitants); rural areas (≤10,000 inhabitants)

**Fig. 1:**
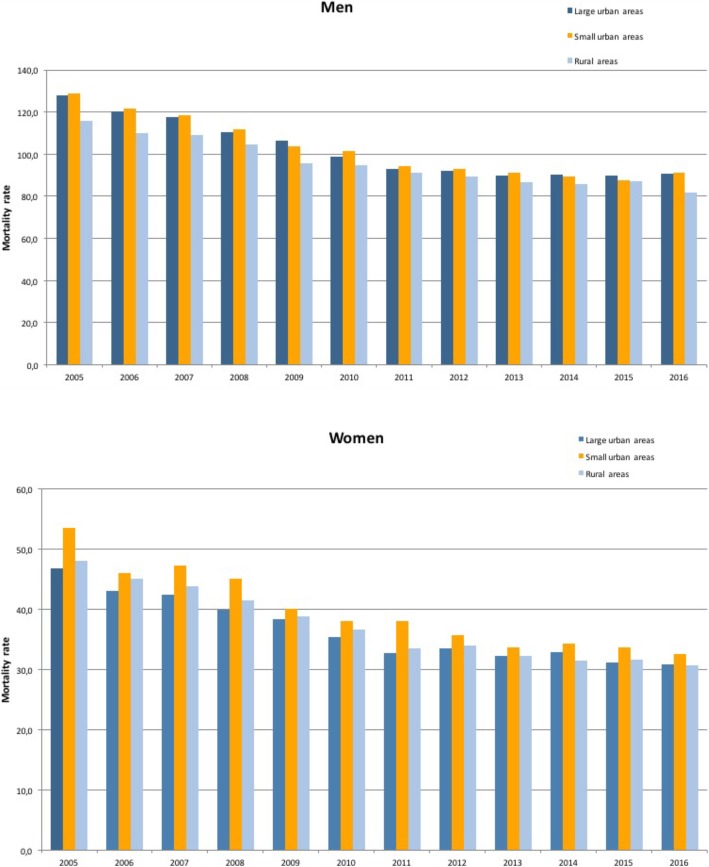
Age-standardized rate of mortality from cardiovascular diseases in people under 75 years of age per 100,000 inhabitants, by area of residence, in men and women. Spain, 2005–2016

In men, those in small urban areas had the highest mortality, and those in rural areas had the lowest (Fig. 1). The mortality rate per 100,000 population in small urban areas and rural areas was 129.0 and 115.6 in 2005, and 81.2 and 82.0 in 2016, respectively. In women, those in small urban areas also had the highest mortality, while those in large urban areas had the lowest. However, beginning in 2012, the mortality rate in residents of large urban areas was similar to that observed for residents in rural areas (Fig. 1). In 2016, the mortality rate per 100,000 population in large urban areas and rural areas was 30.9 and 30.7, respectively.

In men, mortality declined before and during the crisis in all three areas, but after the crisis only the rural areas saw a decline. The greatest reduction in mortality occurred during the first 3 years of economic crisis. In 2008–2010, the mAPC in large urban areas, in small urban areas and in rural areas was, respectively, − 5.7, − 5.6 and − 4.8. And in 2011–2013, the mAPC was − 3.2, − 3.3 and − 2.2 in each of these areas (Table 2). In women, mortality decreased before, during and after the crisis in all three areas; furthermore, the greatest decline occurred in the first 3 years of the crisis and the smallest decline was after the crisis. In 2008–2010, the mAPC in large urban areas, small urban areas and rural areas was, respectively, − 6.0, − 6.0 and − 6.2. And in 2011–2013, the mAPC was − 2.6, − 3.7 and − 3.9, respectively (Table 2).

**Table 2:**
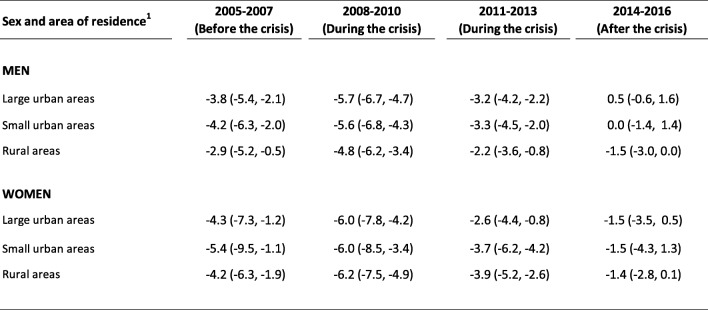
Mean annual percentage change in the age-adjusted (0–74 years) mortality rate (95% confidence interval) from cardiovascular diseases before, during and after the 2008 economic crisis in Spain, by sex and area of residence

^1^Large urban areas (> 100,000 inhabitants and provincial capitals); small urban areas (10,001 to 100,000 inhabitants); rural areas (≤ 10,000 inhabitants)

Table 3 shows the evolution of premature cardiovascular mortality and the mortality rate ratio by area of residence. In men, there were no significant differences in the rate ratios between the two urban areas. In contrast, the mortality ratio in rural areas with respect to large urban areas went from 0.92 [95% confidence interval 0.90–0.94)] in 2005–2007 to 0.97 (0.95–1.00) in 2011–2013 and to 0.94 (0.92–0.96) in 2014–2016. In women, the mortality ratio was higher in small urban areas, but with respect to large urban areas it went from 1.11 (1.08–1.14) in 2005–2007 to 1.06 (1.02–1.09) in 2014–2016. There were no significant differences between mortality in rural areas and large urban areas, although the mortality ratio went from 1.04 (1.00–1.07) in 2005–2007 to 0.99 (0.95–1.03) in 2014–2016.

**Table 3:**
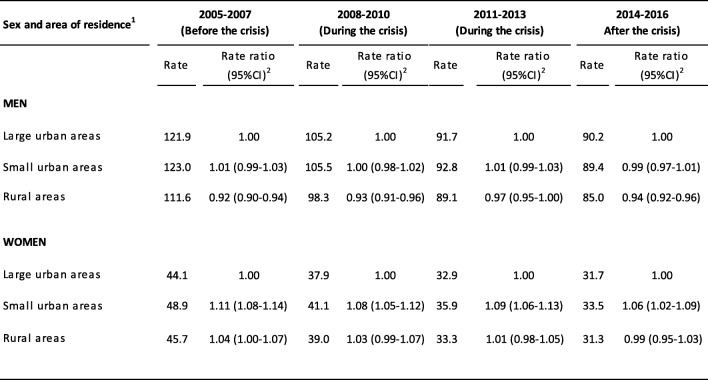
Rate of premature mortality from cardiovascular diseases per 100,000 inhabitants - year and mortality rate ratio before, during and after the 2008 economic crisis in Spain, by sex and area of residence

^1^Large urban areas (> 100,000 inhabitants and provincial capitals); small urban areas (10,001 to 100,000 inhabitants); rural areas (≤ 10,000 inhabitants)

^2^Rate ratio (95% confidence interval)

In general, in all three areas analysed, the prevalence of smoking, obesity and physical inactivity decreased in the study period, except for obesity in men (Table 4). In men, the lowest prevalence of smoking, obesity and physical inactivity was observed in rural areas. Specifically, the percentage ratio in rural areas for smoking in 2006, 2011 and 2016 was 0.99 [95% confidence interval 0.92–1.07], 0.90 (0.83–0.97) and 0.89 (0.83–0.97), respectively. For obesity the respective figures were 0.89 (0.80–0.99), 0.80 (0.71–0.90) and 0.88 (0.79–0.98), and for physical inactivity they were 0.89 (0.86–0.92), 0.89 (0.83–0.95) and 0.86 (0.79–0.92). In women, no significant differences by area of residence were found in the prevalence of smoking or physical inactivity. In contrast, the lowest prevalence of obesity was observed in rural areas, where the percentage ratio in 2006, 2011 and 2016 was 0.86 (0.77–0.96), 0.85 (0.75–0.97) and 0.92 (0.81–1.03), respectively (Table 4).

**Table 4:**
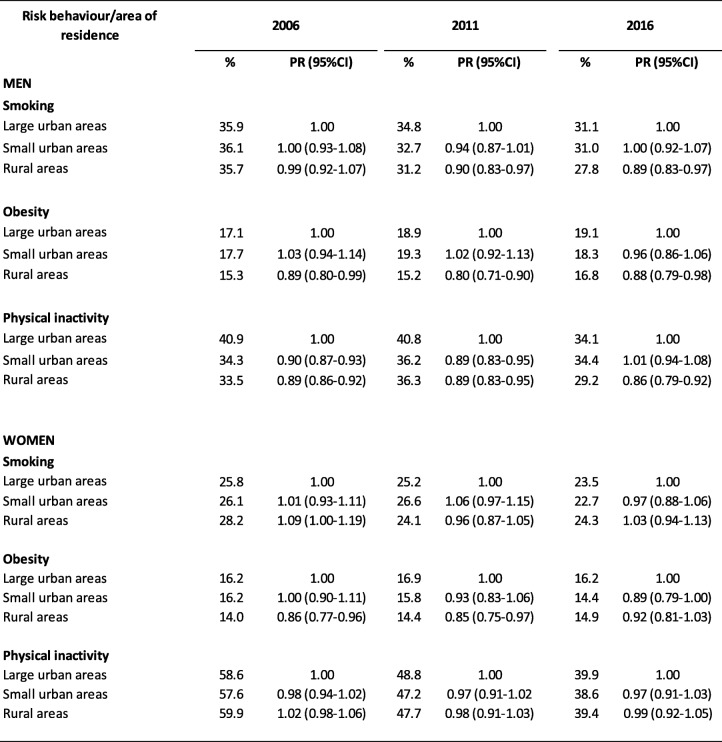
Prevalence of smoking, obesity and sedentarism in the population 15–74 years of age. Spain, 2006, 2011 and 2006. Age-adjusted percentage, percentage ratio (PR) and 95% confidence interval (95%CI), by sex and area of residence

## Discussion

### Main findings

In men, premature cardiovascular mortality was lowest in rural areas. During the economic crisis, mortality declined more in rural than in urban areas and, consequently, by the end of the crisis mortality in rural areas was close to that of urban areas. However, after the crisis, in 2014–2016, mortality by area of residence was similar to what it was before the crisis.

In women, before and during the economic crisis, mortality was slightly higher in rural than in large urban areas, although the lowest mortality was observed in small urban areas. In general, in all three time periods analysed, the mortality decline in rural and small urban areas was larger than in large urban areas. Consequently, in 2014–2016, there were no differences between mortality in large urban and rural areas, although the mortality in small urban areas remained higher.

The pattern of risk behaviours by area of residence was similar in all 3 years analysed. In men, the lowest prevalence of smoking, obesity and physical inactivity was observed in rural areas. In women, obesity was also lowest in rural areas.

### Comparison with other studies and possible explanations

In the USA, Canada, Australia and Sweden, cardiovascular mortality is higher in rural than in urban areas, which has been attributed to a higher prevalence of smoking, physical inactivity and obesity [2–6]. On the other hand, in the various countries of the United Kingdom – England, Wales, Northern Ireland and Scotland – where cardiovascular mortality is higher in urban areas, this has been attributed to increased air pollution and a higher prevalence of smoking in these areas [1, 40, 42].

Our findings in men are similar to those reported in the United Kingdom [1, 40, 42]. Conversely, our findings in women differ from those observed in other countries. According to the Spanish national health surveys, men in rural areas show a lower prevalence of smoking, physical inactivity and obesity, but in women this pattern is only observed in the case of obesity. This different pattern of risk behaviours in men and women could contribute to their differences in cardiovascular mortality by area of residence.

This is the first study that shows the pattern of mortality from cardiovascular diseases and the pattern of risk behaviors in urban and rural areas in Spain. We do not know the reasons for this different pattern in men and women. A possible explanation could be that the socioeconomic profile according to the area of residence was different in women and men. It is known that risk behaviors for health are related to the educational level. However, the educational level in men and women does not show a different distribution depending on the area of residence. Therefore, all we can say is that for unknown reasons, men in rural areas tend to adopt health risk behaviors in a lesser proportion than men living in urban areas, but this does not happen in women.

Several studies have found a greater decline in cardiovascular mortality during economic crises [15, 17, 19, 21]. In our study, this greater decline was observed mainly in the first years of the crisis. Between 2006 and 2011 there was an important reduction in smoking in men and in physical inactivity in women, which could have contributed to the larger decline in mortality at the beginning of the crisis. Between 2006 and 2011, the prevalence of smoking in men decreased from 35.9 to 34.8% in large urban areas, from 36.1% a 32.7% in small urban areas, and from 35.7 to 31.2% in rural areas, while physical inactivity in women declined from 58.6 to 48.8% in large urban areas, from 57.6 to 47.2% in small urban areas, and from 59.9 to 47.7% in rural areas.

In 2014–2016, the decrease in cardiovascular mortality showed a deceleration with respect to the previous periods. The reason for this was twofold: intense influenza virus activity in 2015 and the heat wave in June and July of the same year. The predominant virus type that year was A (H3N2), which was highly lethal and affected both the young and the elderly. As a result, in 2015 deaths due to respiratory and cardiovascular diseases increased in the winter months in Spain, as well as in many other European countries [43]. In addition, cardiovascular deaths in that year increased due to the summer heat wave, which was the second hottest in several decades [44, 45]. The deceleration of the mortality decline was lower in rural areas. The spread of the influenza virus may have been lower in these areas and / or their population may have been better protected from the summer heat. For this reason, the largest decrease in mortality in 2014–2016 was observed in rural areas.

### Strengths and limitations

Mortality data are a source of information of great value, since they collect a phenomenon -death- exhaustively and, in addition, it is a routine source which allows the comparison of the mortality rate over time. However, the analysis by cause of death may be biased if a large percentage of deaths are coded as poorly defined cause of death (codes R00-R99 of the ICD-10). Such a bias does not occur in the mortality data in Spain because only 2% of premature deaths are assigned to those codes [25].

Some cardiovascular deaths are not related to the risk factors analyzed, such as valvular diseases. However, premature deaths from valvular diseases represent only 1% in men and 3% in women of all premature cardiovascular diseases. On the other hand, premature deaths from heart disease, cerebrovascular diseases and hypertension, related to the risk factors studied, represent 93% in men and 95% in women of all premature cardiovascular deaths [25].

This is the first study to provide a description of premature cardiovascular mortality in Spain by degree of urbanization. Furthermore, we have analysed this mortality before, during and after the economic crisis, making it possible to evaluate whether the crisis has altered the pattern of mortality. Previous investigations have analysed cardiovascular mortality in rural and urban areas, but this dichotomy may mask some differences between these areas [46]. Here, we have subdivided urban areas, which has allowed us to detect mortality differences between the two urban areas in women.

It is possible that the economic crisis led to greater modification of cardiovascular mortality in areas with a more economically active population. This has probably had little impact on our results, since there is no difference in the labor force participation rate between the rural and urban areas: 82% in men and 75% in women in both areas were economically active, according to the 2011 population census [47].

Our study, like previous ones conducted in other countries, does not estimate what proportion of the differences in mortality among areas of residence is attributable to behavioural risk factors [2, 48]. Rather, we merely suggest a possible explanation based on the relationship between area of residence and the prevalence of cardiovascular risk behaviours.

Selective migration of subjects with better health to urban areas and increased access to health services in urban areas may contribute to the pattern of cardiovascular mortality according to the degree of urbanization of the area of residence [1, 46]. In our study, we analysed mortality based on residence on the date of death, and the residence of subjects in previous years and/or the place of birth is unknown. Likewise, we do not know the level of access to health services by degree of urbanization. In any case, mortality in both types of urban areas in men, and in small urban areas in women, was higher than mortality in rural areas, which rules out these two explanations.

## Conclusion

In men, the small change in premature cardiovascular mortality by degree of urbanization during the economic crisis was short-lived, since the pattern of mortality was similar before and after the crisis. This pattern is characterized by lower mortality in rural areas. In women, the trend in the mortality rate, even before the economic crisis, modified the pattern of cardiovascular mortality by degree of urbanization of the area of residence observed at the beginning of the period analysed. At the end of the period, the mortality in rural and in large urban areas was similar, and the difference in mortality between the two urban areas was smaller than at the beginning of the period. The different pattern of risk behaviours could explain the different findings in men and women. This different pattern suggests that public health interventions to reduce the burden of cardiovascular disease in the population should establish different priorities for men and women, depending on the rural or urban setting where such interventions are implemented.

## Additional files

Additional file 1: **Table S1.** Population under 75 years of age, by sex and size of municipality of residence. Spain, 2005–2016. (DOCX 17 kb)

Additional file 2: **Table S2.** Deaths from cardiovascular diseases in people under 75 years of age, by sex and size of municipality of residence. Spain, 2005–2016. (DOCX 14 kb)

## Data Availability

The data used in this paper can be obtained freely in the web of the National Statistics Institute (INE): https://www.ine.es

## Abbreviations

BMI: Body mass index
GDP: Gross domestic product
INE: National Institute of Statistics
mAPC: Mean annual percentage change
MRR: Mortality rate tatio
USA: United States of America
