# Supplementary material for: Cardiovascular mortality and risk behaviours by degree of urbanization before, during and after the economic crisis in Spain
Source: BMC Public Health. 2019 Aug 14;19:1109. doi: 10.1186/s12889-019-7427-4 (PMC6694539; doi:10.1186/s12889-019-7427-4)
Supplement: Supplementary file 1 [file 12889_2019_7427_MOESM1_ESM.docx]

Supplementary table 1. Population under 75 years of age, by sex and size of municipality of residence. Spain, 2005-2016

Sex and area of residence[1]	2005	2006	2007	2008	2009	2010	2011	2012	2013	2014	2015	2016
MEN												
Large urban areas	8,573,730	8,650,859	8,644,100	8,875,917	8,989,490	8,985,579	8,991,991	8,955,869	8,896,803	8,771,825	8,733,134	8,715,484
Small urban areas	7,348,946	7,541,223	7,707,671	7,918,119	8,030,036	8,088,179	8,111,613	8,139,466	8,115,710	8,061,936	8,038,762	8,003,683
Rural areas	4,538,966	4,537,399	4,571,163	4,579,990	4,572,402	4,579,684	4,561,070	4,540,344	4,500,685	4,476,857	4,456,459	4,424,804
WOMEN												
Large urban areas	8,832,449	8,892,304	8,895,458	9,101,109	9,220,250	9,240,111	9,273,845	9,255,029	9,219,595	9,119,594	9,101,822	9,095,054
Small urban areas	7,136,700	7,300,884	7,470,431	7,656,076	7,781,290	7,855,009	7,893,106	7,935,946	7,938,619	7,909,428	7,905,105	7,880,676
Rural areas	4,199,444	4,188,194	4,213,305	4,199,508	4,199,976	4,210,905	4,192,185	4,168,882	4,143,400	4,128,608	4,114,952	4,085,105

1. Large urban areas (> 100,000 inhabitants and provincial capitals); small urban areas (10,001 to 100,000 inhabitants); rural areas (≤ 10,000 inhabitants)
