# Supplementary material for: Cardiovascular mortality and risk behaviours by degree of urbanization before, during and after the economic crisis in Spain
Source: BMC Public Health. 2019 Aug 14;19:1109. doi: 10.1186/s12889-019-7427-4 (PMC6694539; doi:10.1186/s12889-019-7427-4)
Supplement: Supplementary file 2 [file 12889_2019_7427_MOESM2_ESM.docx]

Supplementary table 2. Deaths from cardiovascular diseases in people under 75 years of age, by sex and size of municipality of residence. Spain, 2005-2016

**Sex and area of residence[1]**	**2005**	**2006**	**2007**	**2008**	**2009**	**2010**	**2011**	**2012**	**2013**	**2014**	**2015**	**2016**
MEN												
Large urban areas	8,600	8,231	8,070	7,751	7,537	7,012	6,734	6,714	6,677	6,787	6,974	7,100
Small urban areas	6,930	6,763	6,733	6,519	6,109	6,028	5,766	5,762	5,774	5,737	5,834	6,127
Rural areas	4,990	4,693	4,642	4,378	3,949	3,893	3,759	3,660	3,578	3,633	3,784	3,599
WOMEN												
Large urban areas	3,810	3,532	3,482	3,313	3,190	2,931	2,775	2,834	2,775	2,868	2,830	2,832
Small urban areas	3,115	2,744	2,866	2,769	2,490	2,376	2,418	2,283	2,196	2,273	2,324	2,267
Rural areas	2,197	2,029	1,947	1,795	1,652	1,508	1,378	1,363	1,291	1,289	1,335	1,292

1. Large urban areas (> 100,000 inhabitants and provincial capitals); small urban areas (10,001 to 100,000 inhabitants); rural areas (≤ 10,000 inhabitants)
